# Pre-exercise health screening in the UAE: A necessity or barrier to engage in physical activity?

**DOI:** 10.1371/journal.pone.0325246

**Published:** 2025-05-30

**Authors:** Rifah Anwar Assadi, Afreen Abdul Rahim Sanaullah, Sathyapriya Nandagopal, Shahnaz Mohamed Wazil, Priya Pardasani, Meenadarsini Dhamothiran, And Ozyigit, Jagat Gopinath, Ans Ahmed Mahmood, Maryam Almarzooqi, Bibi Morsal Sayedy, Elham Riyaz, Jayakumary Muttappallymyalil

**Affiliations:** 1 Gulf Medical UniversityMaster of Public Health (MPH) Program, College of Medicine, Gulf Medical University, Ajman, United Arab Emirates; 2 Gulf Medical UniversityDoctor of Medicine (MD) Program, College of Medicine, Gulf Medical University, Ajman, United Arab Emirates,; 3 Bachelor of Medicine, Bachelor of Surgery (MBBS) Program, College of Medicine, Gulf Medical University, Ajman, United Arab Emirates,; 4 Higher Diploma in Preclinical Sciences (HDPCS) Program, College of Medicine, Gulf Medical University, Ajman, United Arab Emirates,; 5 Department of Community Medicine, College of Medicine, Gulf Medical University, Ajman, United Arab Emirates; RAK Medical and Health Sciences University: Ras Al Khaimah Medical and Health Sciences University, UNITED ARAB EMIRATES

## Abstract

**Background:**

Sedentary lifestyles contribute to the rise of non-communicable diseases, making physical activity (PA) crucial for public health. Pre-exercise screening is an important tool for ensuring safety, but its utilization and the factors influencing its adoption need further exploration.

**Objective:**

This study assesses the utilization of pre-exercise screening among physical activity facility users in the UAE, identifying sociodemographic and health-related factors associated with screening practices.

**Methods:**

A cross-sectional study was conducted among adults aged 18 and above in the UAE. Data were collected through a self-administered questionnaire from 630 adults using PA facilities, covering socio-demographic characteristics, PA engagement, knowledge of PA benefits, and pre-exercise screening practices. Data analysis was performed using SPSS version 28 for descriptive and inferential statistics.

**Results:**

Of the participants, 496 (78.7%) were unemployed, 554 (87.6%) were aged 18–34, 294 (46.7%) had bachelor’s degrees, and 522 (82.9%) were single. Females made up 52% of the respondents. Only 186 (29.5%) underwent pre-exercise screening, with 377 (59.8%) not screened and 67 (10.6%) uncertain. Associations were found between higher screening utilization and factors such as being over 30 years old (44.9%), male (33.9%), having higher education (33.5%), and being employed (40.3%). Participants with chronic health conditions, including heart disease (52.4%), chest pain (48%), and mental health problems (50%), were significantly more likely to utilize pre-exercise screening (P < 0.001). The purpose of pre-exercise screening as risk stratification was recognized by 214 (34.1%), while 257 (40.7%) understood its preventive role. Using the 2023 PARQ + , 401 (63.7%) were cleared for PA, and 229 (36.3%) required further evaluation due to medical or mental health issues. Most participants (83.4%) did not receive guidance from exercise professionals, but 74.3% favoured mandatory pre-exercise screening.

**Conclusions:**

The study highlights a gap in pre-exercise screening utilization in the UAE, with significant associations to sociodemographic factors and health conditions. The findings highlight the need for increased awareness and adoption of pre-exercise screening in the UAE. Addressing knowledge gaps and implementing mandatory screening protocols could improve health literacy and safety in PA facilities.

## Introduction

According to World Health Organization, globally around 1.4 billion adults are physically inactive. Around one-fourth of men and one-third of women worldwide do not engage in sufficient physical activity to maintain their health. WHO describes physical activity “as any bodily movement produced by skeletal muscles that requires energy expenditure” [1]. Exercise influences cognitive functioning in the brain and well-being in regard to mental and physical health [2]. A minimum of between 150–300 minutes of moderate physical activity per week or 75–150 minutes of intense physical activity per week is recommended in order to avoid weight gain, increase weight loss and boost physical fitness [3]. Physical activities include walking, sports, cycling, running, weight-lighting, stretching exercises [1]. Lack of physical activity is known to be a modifiable risk factor for cardiovascular disease and chronic illnesses that includes obesity, cancer, diabetes, hypertension, depression, osteoarthritis and osteoporosis [4]. A study conducted among 76 countries between 2002 and 2004, found that around 21.4% are not physically active [5]. In 2016, 27.5% of adults worldwide were physically inactive [6]. In the MENA and Central Asia regions, 32.8% adults are physically inactive [7]. In Arab countries, the prevalence of physical exercise varied from 34.2 to 96.9% [8]. In the UAE adult population, around 58% are physically inactive [9]. From 2017–2021, 19% school children in UAE obtained the necessary level of moderate to vigorous physical activity [10]. According to DCD survey in Abu Dhabi, around 37.7% respondents met recommendations of WHO for physical activity [11].

Regular physical exercise reduces the risk of cardiovascular diseases for both women and men [12], but it’s also known for exercise related deaths in asymptomatic individuals from the rupture of atherosclerotic plaque in a coronary artery, leading to acute thrombosis [13,14]. In sports related to male marathon runners, mostly cardiac arrest is often linked to atherosclerotic coronary artery disease or hypertrophic cardiomyopathy [15]. Muscle injuries are the most prevalent type of injury in sports, accounting for 10–55% of all the injuries [16]. Improved fitness levels can help slow down the progression toward hypertension [17]. For certain individuals, engaging in higher-intensity exercise may yield additional health benefits, whereas others might experience greater advantages from lower-intensity activities performed over a longer duration [18]. According to ACSM (American College of Sports Medicine) guidelines for exercise testing and prescription, exercise related event risk like sudden cardiac death or Acute Myocardial infarction is higher in individuals with factors of vigorous intensity PA, and/ or unaccustomed PA [19]. The American College of Sports Medicine recommends incorporating resistance training as a supplementary component to an aerobic-based exercise program to help reduce blood pressure [20]. To ensure clarity for patients, minimize the risk of injury and maintain the effectiveness of exercise. The healthcare providers should approach exercise as a carefully prescribed intervention, as related to medications.

Pre-exercise screening is carried out to identify individuals who may have conditions that increase their risk of experiencing a negative outcome during physical exercise or activity. It is a safety net that helps to assess if the potential benefits of exercise outweigh the hazards for an individual [21]. In 2010, three national organizations- ESSA (Exercise and Sports Science Australia), SMA (Sports Medicine Australia) and FA (Fitness Australia) collaborated to standardize pre-exercise screening in the Australian health and fitness industry. Many fitness industries have used several screening tools which are adopted from international organizations like the Canadian PAR-Q or the American College of Sports Medicine’s risk stratification system. In 2011, three Australian organizations created the Adult Pre-Exercise Screening System (APSS) and its basic equipment, the Tool (APSS Toll) [22]. The ACSM recommendations have replaced the risk factor evaluation with an algorithm that stratifies first by present physical activity level, then by chronic illness presence and/or symptoms and then finally by desired exercise intensity [23]. Despite its importance, there are significant gaps in the implementation and awareness of effective screening protocols worldwide. This disparity is evident in various regions, particularly in developing countries, where financial constraints and lack of knowledge hinder the adoption of standardized practices.

## Methods

The research aims to explore the utilization of pre-screening among physical activity facilities users in Ajman, UAE, through a cross-sectional study design. It targets adult users of such facilities, focusing on those who are registered, over 18 years old, of any gender or nationality, and excludes those unwilling to participate. A sample size of 426 participants is calculated based on existing literature [24], factoring in a nonresponse rate and utilizing convenience sampling for recruitment.

The study employed a self-administered questionnaire to gather data on socio-demographic characteristics, literacy on physical activity, and practice details including type, frequency, and duration of physical activity, as well as use of pre-exercise screening from 17 June 2023–19 February 2024. This instrument underwent face and content validation by experts, followed by a pilot test.

Ethical considerations for the study included obtaining approval from the Gulf Medical University Institutional Review Board (IRB-COM-STD-103-May-2023), ensuring participant consent, maintaining anonymity, and upholding voluntary participation, with no risk to participants. The study proceeded with IRB approval, obtained permissions from relevant facilities, and data collection was carried out using an online Google questionnaire form. This method was chosen for its feasibility, considering the short response time required, lack of harm to participants. Consent was obtained digitally through participants’ explicit selection of “Yes” or “No” to indicate their agreement to participate in the online questionnaire. The consent process was witnessed by the data collection team to ensure transparency and compliance.

Data will be securely stored for three years and analyzed using SPSS for descriptive and inferential statistics, with significance set at a p-value ≤ 0.05.

## Results

A total of 630 responses were collected from adults using physical activity facilities in the UAE. Table 1 presents the demographic characteristics of the participants. Among them, 496 (78.7%) were unemployed, and 134 (21.3%) were employed. The gender distribution was relatively balanced, with 329 (52.2%) females and 301 (47.8%) males. The majority of participants were aged 30 years or older (527; 83.7%), and most were single (522; 82.9%). Regarding education, 409 participants (64.9%) had education beyond high school, 210 (33.3%) had a high school education, and only 11 (1.7%) had education below high school level.

**Table 1:** Distribution of sociodemographic variables.

Sociodemographic	Variable	No.	Percent
Employment Status	Employed	134	21.3
	Unemployed	496	78.7
Gender	Female	329	52.2
	Male	301	47.8
Age Category	≤30 years	532	84.4
	>30 years	98	15.6
Marital Status	Single	522	82.9
	Married	108	17.1
Highest Educational Level	Below High School	11	1.7
	High School	210	33.3
	Above High School	409	64.9

**Fig 1:**
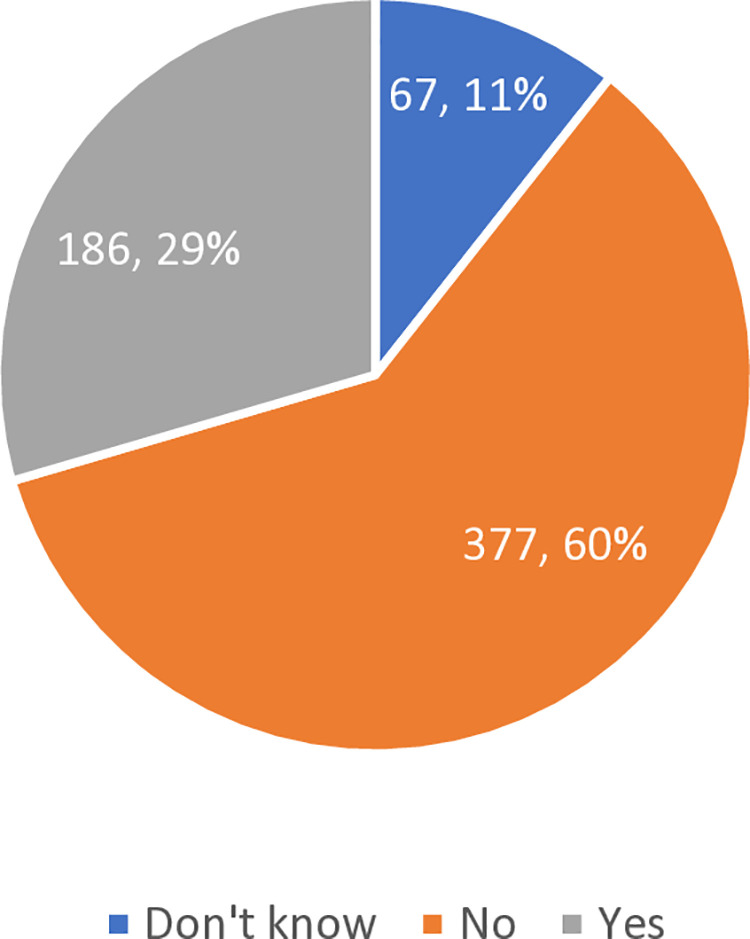
Distribution of participants according to self-reported pre-exercise screening utilization.

Fig 1 illustrates the self-reported utilization of pre-exercise screening among participants. Of the 630 respondents, 377 (60%) reported not utilizing pre-exercise screening, 186 (30%) indicated they utilized it, and 67 (11%) were unsure of their use of pre-exercise screening.

Table 2 summarizes the association between pre-exercise screening utilization and participants’ sociodemographic characteristics. Significant associations were found for employment status (P < 0.01), gender (P < 0.05), age category (P < 0.01), and educational level (P < 0.05), while marital status showed no significant relationship.

**Table 2:** Association between pre-exercise screening utilization and Sociodemographic characteristics.

Variable	Group	Utilization of Pre-Exercise screening						Total	P
		Yes		No		Don’t Know			
		No.	%	No.	%	No.	%		
Employment Status	Employed	54	40.3	69	51.5	11	8.2	134	<0.01
	Unemployed	132	26.6	308	62.1	56	11.3	496	
Gender	Female	84	25.5	212	64.4	33	10	329	<0.05
	Male	102	33.9	165	54.8	34	11.3	301	
Age Category	≤30 years	142	26.7	335	63	55	10.3	532	<0.01
	>30 years	44	44.9	42	42.9	12	12.2	98	
Marital Status	Single	144	27.6	323	61.9	55	10.5	522	NS
	Married	42	38.9	54	50	12	11.1	108	
Highest Educational Level	Below High School	2	18.2	7	63.6	2	18.2	11	<0.05
	High School	47	22.4	141	67.1	22	10.5	210	
	Above High School	137	33.5	229	56	43	10.5	409	

NS – not significant.

Employed participants were more likely to have undergone screening (40.3%) compared to unemployed participants (26.6%). Males reported higher screening rates (33.9%) than females (25.5%). Participants over 30 years old had notably higher utilization (44.9%) than those aged 30 or younger (26.7%). Screening rates also increased with higher educational attainment, with those above high school reporting the highest utilization (33.5%).

These findings highlight the influence of sociodemographic factors on engagement with pre-exercise health screenings and point to the need for targeted awareness efforts among lower-participating groups.

S1 Table shows participants’ responses to the General Health Questions of the Physical Activity Readiness Questionnaire (PAR-Q+). Among participants, the most commonly reported health issues were dizziness or loss of balance (18.4%), musculoskeletal problems (15.9%), chest pain (15.6%), heart conditions or high blood pressure (13.3%), other chronic conditions (11.7%), use of prescribed medications (10.8%), and advice to engage only in medically supervised physical activity (8.9%).

Table 3 displays the association between participants’ responses to the PAR-Q+ general health questions and their utilization of pre-exercise screening. Across all seven health-related questions, statistically significant associations were observed (all P < 0.001).

**Table 3:** Association between pre-exercise screening utilization and PARQ+ General health questions.

General Health Questions		Utilization of Pre- Exercise Screening						Total	P
		Yes		No		Don’t know			
		No.	%	No.	%	No.	%		
Has your doctor ever said that you have a heart condition OR high blood pressure?	No	142	26	345	63.2	59	10.8	546	<0.001
	Yes	44	52.4	32	38.1	8	9.5	84	
Do you feel pain in your chest at rest, during your daily activities of living, OR when you do physical activity?	No	139	26.1	339	63.7	54	10.2	532	<0.001
	Yes	47	48	38	38.8	13	13.3	98	
Do you lose balance because of dizziness OR have you lost conscious in the last 12 months?	No	127	24.7	334	64	53	10.3	514	<0.001
	Yes	59	50.9	43	37.1	14	12.1	116	
Have you ever been diagnosed with another chronic medical condition (Other than heart disease or high blood pressure)?	No	146	26.3	354	63.7	56	10.1	556	<0.001
	Yes	40	54.1	23	31.1	11	14.9	74	
Are you currently taking prescribed medications for a chronic medical condition?	No	150	26.7	357	63.5	55	9.8	562	<0.001
	Yes	36	52.9	20	29.4	12	17.6	68	
Do you currently have (or have had within past 12 months) a bone, joint, or soft tissue (muscle, ligament, or tendon) problem that could made worse by becoming more physically active?	No	143	27	335	63.2	52	9.8	530	<0.001
	Yes	43	43	42	42	15	15	100	
Has your doctor ever said that you should only do medically supervised physical activity?	No	149	26	361	62.9	64	11.1	574	<0.001
	Yes	37	66.1	16	28.6	3	5.4	56	

Participants who reported health conditions—including heart conditions or high blood pressure (52.4%), chest pain (48.0%), dizziness or loss of consciousness (50.9%), other chronic conditions (54.1%), prescribed medications (52.9%), musculoskeletal issues (43.0%), and recommendations for medically supervised activity (66.1%)—were significantly more likely to have undergone pre-exercise screening than those who did not report these conditions.

These findings suggest that individuals with existing or perceived health risks are more inclined to participate in pre-exercise screening, underscoring the role of perceived medical vulnerability in health behavior decisions.

**Fig 2:**
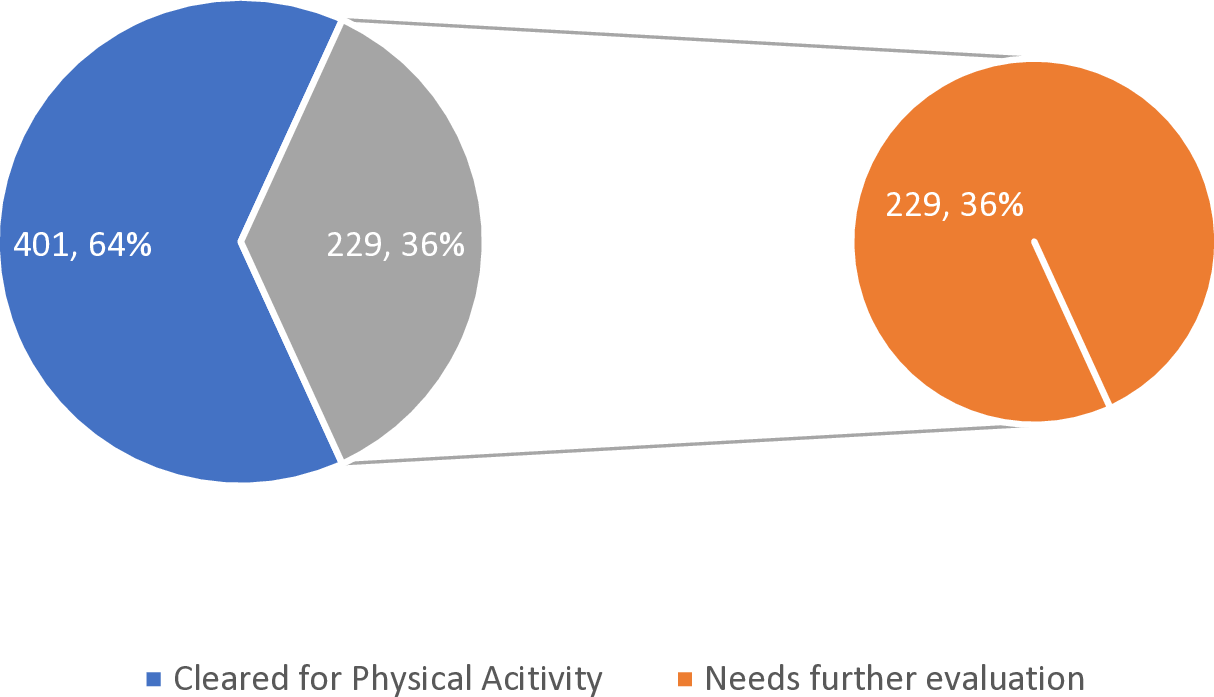
Results of General Health Assessment based on Physical Activity Readiness Questionnaire for everyone (PAR-Q+).

Fig 2 depicts the results of the General Health Assessment based on the PAR-Q+ responses. Of the 630 participants, 401 (64%) were cleared for physical activity, while 229 (36%) requiring further evaluation due to medical conditions or health concerns.

S2 Table shows that the most common follow-up health issues were arthritis, osteoporosis, or back problems (11.4%), followed by mental health or learning difficulties (6.8%), metabolic conditions (6.1%), other or multiple conditions (7.7%), respiratory diseases (5.2%), spinal cord injuries (4.2%), and stroke (3.4%).

Table 4 shows the association between pre-exercise screening utilization and participants’ responses to the follow-up health questions of the PAR-Q + . Significant associations (P < 0.05 or lower) were observed for all listed conditions.

**Table 4:** Association between pre-exercise screening utilization and PARQ+ follow-up health questions.

Follow Up Questions (Further evaluation)		Utilization of Pre-Exercise Screening						Total	P
		Yes		No		Don’t Know			
		No.	%	No.	%	No.	%		
Do you have Arthritis, Osteoporosis, or Back Problems?	Yes	26	40.6	30	46.9	8	12.5	64	<0.05
	No	132	26.6	310	62.5	54	10.9	496	
Do you currently have Cancer of any kind?	Yes	16	72.7	4	18.2	2	9.1	22	<0.001
	No	142	26.4	336	62.5	60	11.2	538	
Do you currently have High Blood Pressure?	Yes	15	60	5	20	5	20	25	<0.001
	No	143	26.7	335	62.6	57	10.7	535	
Do you have a Heart or Cardiovascular Condition? This includes Coronary Artery Disease, Heart Failure, Diagnosed Abnormality of Heart Rhythm	Yes	15	62.5	7	29.2	2	8.3	24	<0.01
	No	143	26.7	333	62.1	60	11.2	536	
Do you have any Metabolic Conditions? This includes Type 1 Diabetes, Type Diabetes, Pre- Diabetes	Yes	18	52.9	14	41.2	2	5.9	34	<0.01
	No	140	26.6	326	62	60	11.4	526	
Do you have any Mental Health Problems or Learning Difficulties?	Yes	19	50	12	31.6	7	18.4	38	<0.001
	No	139	26.6	328	62.8	55	10.5	522	
Have you had a Stroke? This includes Transient Ischemic Attack (TIA) or Cerebrovascular Accident	Yes	14	73.7	4	21.1	1	5.3	19	<0.001
	No	144	26.6	336	62.1	61	11.3	541	
Do you have a respiratory disease?	Yes	16	55.2	11	37.9	2	6.9	29	<0.01
	No	142	26.7	329	62	60	11.3	531	
Do you have a Spinal Cord Injury?	Yes	16	72.7	3	13.6	3	13.6	22	<0.001
	No	129	26	312	62.9	55	11.1	496	
Do you have any other medical condition not listed above or do you have two or more medical conditions?	Yes	21	48.8	15	34.9	7	16.3	43	<0.01
	No	137	26.5	325	62.9	55	10.6	517	

Participants reporting specific medical conditions—such as spinal cord injury (72.7%), stroke or TIA (73.7%), cancer (72.7%), high blood pressure (60.0%), heart or cardiovascular disease (62.5%), and respiratory conditions (55.2%)—had substantially higher rates of pre-exercise screening utilization compared to those without these conditions. Elevated screening rates were also noted among individuals with metabolic conditions (52.9%), mental health problems or learning difficulties (50.0%), arthritis or back problems (40.6%), and those with multiple or unlisted medical conditions (48.8%).

These findings indicate that individuals with more serious or multiple health conditions are significantly more likely to undergo pre-exercise screening, reflecting greater awareness or healthcare referral among higher-risk groups.

**Fig 3:**
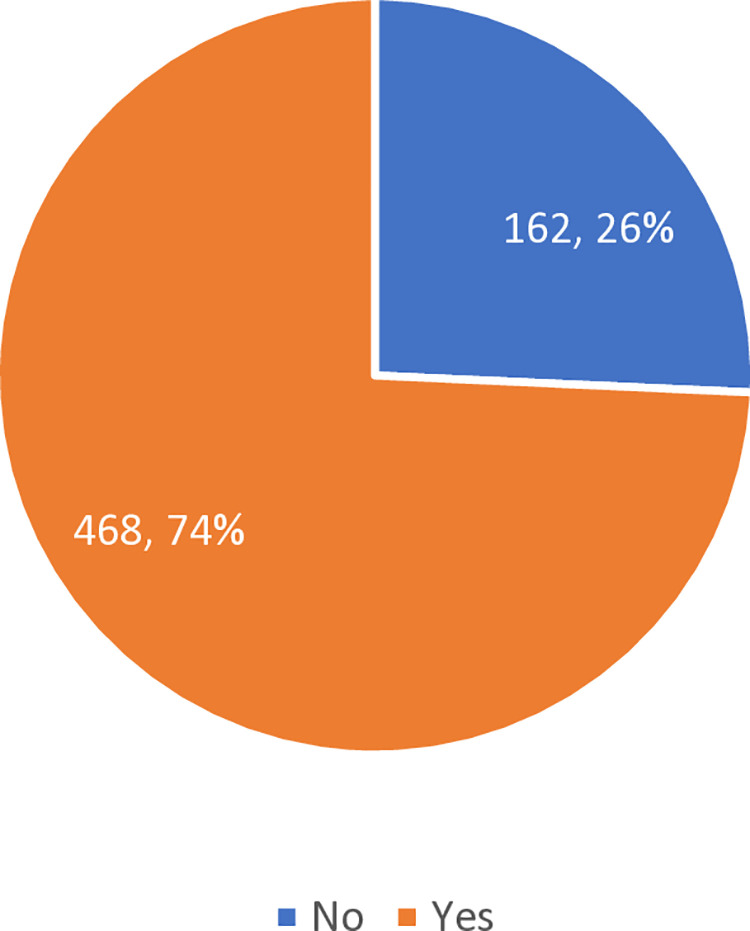
Participants’ stance on making pre-exercise screening mandatory.

Fig 3 highlights the participants’ opinions on mandatory pre-exercise screening. A significant majority, 468 (74%), were in favor of making pre-exercise screening mandatory, while 162 (26%) were opposed.

These findings emphasize the need for increased awareness of pre-exercise screening practices and suggest that mandatory screening could potentially improve safety and health outcomes for individuals engaging in physical activity.

Table 5 presents the association between participants’ stance on making pre-exercise screening mandatory and their actual utilization of such screenings. A statistically significant association was found (P < 0.05). Participants who supported mandatory screening were more likely to have undergone pre-exercise screening (32.7%) compared to those who opposed it (20.4%), while those who opposed the mandate also reported the highest rate of non-utilization (68.5%). These results suggest that positive attitudes toward policy-level screening requirements may be linked to greater personal engagement in screening behavior.

**Table 5:** Association between pre-exercise screening utilization and Participants’ Stance on Making Pre-Exercise Screening Mandatory.

Participants’ Stance on Making Pre-Exercise Screening Mandatory	Utilization of Pre-Exercise Screening						Total	P
	Yes		No		Don’t Know			
	No.	%	No.	%	No.	%		
No	33	20.4	111	68.5	18	11.1	162	<0.05
Yes	153	32.7	266	56.8	49	10.5	468	

## Discussion

Demographic factors play a significant role in shaping health practices. Abd-Allatif conducted a comparative study on physical activity among UAE migrants, which revealed trends similar to those found in our study. However, their findings highlighted key differences, particularly in terms of employment status and health risks. Our, UAE participant facility users had high unemployment rates (see Table 1), while the migrant study participants were all employed, with many working in either physically demanding or sedentary jobs [25]. While high unemployment may limit access to fitness resources among UAE participant facility users, migrant workers face health risks tied to the physical demands of their jobs. Notably, employed participants in our study (40.3%) were more likely to undergo pre-exercise screening compared to unemployed participants (26.6%) (P < 0.01) (see Table 2). Thus, socioeconomic factors influence both groups, but in opposite ways: one group faces economic obstacles, while the other faces occupational health hazards.

Beyond socioeconomic factors, age emerged as another significant influence on physical activity behaviors, with notable differences observed between the younger and older participants. Our study, participant facility users were predominantly young, with 84.4% under 30 (Table 1), while over age 30 (44.9%) showed significantly higher pre-exercise screening utilization (P < 0.01) (Table 2). Young age group is typically more open to physical activity, which contrasts with other study done in UAE among the older migrant cohort [25]. In our study, female consists of 52.2%, while male 47.8% in contrasts with migrant study where participants averaged 34 years for males and 33.6 years for females. Despite their youth, the migrant group faced barriers, such as long working hours and limited leisure time, which hindered their ability to engage in regular exercise [25]. Additionally, Males (33.9%) utilized pre-exercise screening more than females (25.5%) (P < 0.05) (Table 2).

Marital status also had implications for exercise habits. Among participant facility users, 82.9% were single, indicating fewer family responsibilities and more time for exercise. This pattern aligns with the migrant study, which found that single individuals living in shared accommodations tended to engage more in physical activity [25]. Such findings highlight how family obligations and cultural expectations can influence fitness priorities. This is particularly true for women across diverse global settings. In the UAE, cultural expectations often dictate family roles and responsibilities, which can limit opportunities for women to engage in physical activity due to pressure to prioritize family over personal health.

Educational attainment revealed 64.9% of UAE participant facility users had post-secondary education (Table1), while it’s noted that higher education levels in migrant UAE study correlated with physical inactivity levels [25]. Although Higher education was associated with greater screening utilization, with participants above high school (33.5%) more likely to use screening than those with lower education levels (P < 0.05) (Table 2). This demonstrates that, despite education, financial and social factors may be stronger determinants of health behaviour.

The findings underscore significant challenges impacting both pre-exercise screening and physical activity participation. Addressing these issues requires tailored interventions that account for socio-economic, occupational, and cultural factors. Comparative analysis between the two groups can further inform the development of targeted, stratified interventions to effectively meet their distinct needs.

While demographic factors provide context for physical activity habits, the issue of pre-exercise screening is equally critical. The data presented in Fig 1 highlights a concerning 60% non-utilization rate for pre-exercise screenings, with 30% of participants reported the use and 10% unaware of its existence. This finding reveals significant gaps in both health awareness and institutional support in the UAE. These findings align with Herbert, reported only 18% using preparticipation screening in U.S. university fitness facilities [26] while our study indicates an even higher level of disengagement. Furthermore, the 10% of participants unaware of screening highlights a global challenge in effectively communicating health protocols. In Australia, a nationwide self-reported survey of fitness professionals on use of Adult pre exercise screening system (APSS) found that only 20% used the screening extremely frequently, while 45% used it infrequently [27]. Institutional readiness appears to be a key differentiator. While the UAE’s 30% utilization rate demonstrates relatively higher individual compliance, it also highlights systemic shortcomings. Moving forward, transitioning the responsibility from individuals to institutions—by making screenings mandatory and integrated into the membership process for all members—could significantly improve overall health outcomes.

Key health issues that influence physical activity preparedness are highlighted in S1 Table, which displays the PAR-Q+ data. These included dizziness (18.4%), joint problems (15.9%), chest pain (15.6%), cardiac ailments or hypertension (13.3%), chronic diseases (11.7%), and medication use (10.8%). In our study about 18.4% reported dizziness, while such conditions are known to effect adults 15% to 20% yearly with prevalence increasing with age [28]. Randomized controlled trial of 6 month concluded Supervision of Physical therapy guided intervention such as supervised Vestibular rehabilitation therapy (VRT) is highly effective for subjective dizziness with chronic peripheral vestibular disorders [29]. Remarkably, 15.6% of participants reported experiencing chest pain, which is a crucial sign that these people could require adapted exercise regimens, including low-impact exercises, to reduce joint and heart strain. The significance of pre-exercise screenings in identifying high-risk individuals who might need medical examinations or customised exercise regimens to safely participate in physical activity is highlighted by these findings. Globally, similar barriers to physical activity have been reported. For instance, Alsaleh and Baniyasin found that 56.7% of coronary heart disease (CHD) patients in neighbouring Jordan had low physical activity due to discomfort, reflecting the joint issues and chronic conditions observed in our study [30]. In our study 13.3% reported with cardiac ailments or hypertension following 11.7% with chronic diseases. Similarly, a study by Bytyci Katanolli in Kosovo found that individuals with diabetes and hypertension often avoid physical activity due to fear of worsening their health [31]. Fitness professionals and facilities should prioritize strategies like promoting isometric exercise training (IET) as it’s known as antihypertensive alternative [32] with high adherence & 11.2–12.9 mmHg reductions in office systolic BP of healthy adults [33] and expanding healthcare access. These approaches can foster inclusive, evidence-based programs that enhance physical activity readiness and adherence, even in resource-constrained settings.

While our study noted significant association for utilization of pre-exercise screening (see Table 3) with a history of heart conditions or high blood pressure (52.4%) (P < 0.001), participants reporting chest pain (48%), balance issues (50.9%), or other chronic medical conditions (54.1%) (P < 0.001). Also, Use of prescribed medications (52.9%) and musculoskeletal issues (43%) significantly correlated with increased pre-exercise screening utilization (P < 0.001).

Fig 2 that shows 64% of participants were cleared for physical activity, while 36% required further evaluation. But according to UAE National Health Survey 2017–2018 reported that 67.9% of adults aged 18–69 years were overweight (BMI ≥ 25 kg/m2) [34]. While a study among US adults aged 40 years had a notable over 90% recommendation for physician consult by using American Heart Association/American College of Sports Medicine’s Preparticipation Questionnaire (AAPQ) in addition to similar results using Physical activity readiness questionnaire (PAR-Q) [35]. These figures suggest that more attention needs to be paid to the underlying health issues that may prevent full participation in exercise. Following on responses to PARQ+ questions to 36% that were not cleared for PA at initial screening (See S2 Table), 64 (11.4%) of participants reported having Arthritis, Osteoporosis, or Back Problems. Approximately 1.71 billion people worldwide live with musculoskeletal conditions [36]. According to evidence stated by College of Family Physicians of Canada Physical activity series – with a favourable risk to benefit ratio for patients with these conditions, also stating adverse events risk is somewhat higher in specific category of patients thereby emphasis individualized approach [37].

In our study, 38 (6.8%) participants reported having mental health problems or learning difficulties. One study found the prevalence of UAE adults suffering from at least one mental health disorder to be 57.2%, with Anxiety and depression being the most common conditions [38]. A systematic review and network meta-analysis including 218 different studies found that physical activity was an effective treatment for depression, with walking or jogging, yoga, and strength training more effective than other exercises [39].

Merely 34 (6.1%) of the individuals indicated that they had metabolic problems, such as type 1 or type 2 diabetes. A cross-sectional study found the overall prevalence of metabolic syndrome in the UAE to be 37.4% [40], While 16.4% is reported as age adjusted diabetes prevalence in adults (20–79 years) by Diabetes Atlas (International Diabetes Federation- IDF) [41]. The American Diabetes Association has concluded in its position statement that exercise and physical activity should be advised and incorporated into the care plan for all individuals with diabetes to help manage blood sugar levels and promote overall health. Additionally, it lists various groups with specific exercise type, intensity and structuring in accordance with conditions [42].

With our study, second most common heath issue reported condition other than the listed or to having two or more conditions of about 7.7%. Apart from the listed were respiratory disease (5.2%), Cardiovascular conditions (coronary artery disease, heart failure or Rhythm abnormality) (4.3%), spinal cord injury (4.2%), Cancer of any kind (3.9%), stroke (which included Transient Ischemic Attack (TIA) or Cerebrovascular Accident (3.4%). In Physical activity practice guideline for Americans, disability or chronic health conditions individuals are recommended as per their ability and avoid inactivity, and highlighted to be under care of healthcare provider, additional consult for guidance on amount and type of activity [43]. As seen the participants in our study in Table 4, showing higher screening rate with cancer (72.7%), high blood pressure (60%), or cardiovascular conditions (62.5%) (P < 0.001), while those with mental health problems (50%), strokes (73.7%), respiratory diseases (55.2%), and spinal cord injuries (72.7%) were more likely to undergo pre-exercise screening (P < 0.01 to P < 0.001).

Notably, about 74% were in favour of making pre- screening mandatory with about 32.7% (of 468) utilizing the screening service and 20.4% (of 162) of opposed not in favour also utilizing if made mandatory (see Table 5). But recent guidelines, such as those from the American College of Sports Medicine (ACSM), have moved towards a more liberal approach as seen in review conducted for community lifestyle intervention program [44]. ACSM states sedentary individuals can commence light to moderate intensity exercise program, and that all individuals should participate in preparticipation screening for exercise. For general population, use of an exercise professional via an algorithm before initiating a moderate to vigorous program by using Physical Activity Readiness Questionnaire Plus (PAR-Q+) [45]. The goal of these revised guidelines is to lower obstacles to exercise engagement while considering the patient’s medical history, intended exercise intensity, current level of physical activity, and the existence of CVD signs and symptoms [45].

## Conclusions

Overall, the study reveals that only one-third of participants (186 out of 630) utilized pre-exercise screening before engaging in physical activity. Despite the underutilization, the findings highlight that several factors—such as sociodemographic characteristics, chronic health conditions, and attitudes toward mandatory screening—play a critical role in the adoption of pre-exercise screening. Notably, while all participants were exposed to screening, approximately two-thirds (401 out of 630) were cleared for physical activity, with the remaining one-third (229 out of 630) requiring further evaluation due to health concerns. Addressing knowledge gaps, implementing policies that support mandatory screening, and shifting public attitudes could enhance the uptake of this preventive measure, ultimately improving health safety during physical activity. Additionally, the study finds a significant association between participants’ stance on making pre-exercise screening mandatory and its actual utilization, emphasizing the influence of personal attitudes on screening practices.

Physical activity is a cornerstone of overall health and well-being, offering a myriad of benefits that extend far beyond physical fitness. Regular exercise can lead to significant improvements in cardiovascular health, diabetes management, cancer prevention, and mental health [46]. For optimal results, exercise prescriptions should be personalized, considering factors such as the specific chronic condition, current fitness level, and individual goals. These findings emphasize the need for greater awareness and implementation of pre-exercise screening, as many individuals may have underlying conditions that could affect their ability to safely participate in physical activity in terms of intensity, frequency, duration and mode.

Various forms of exercise and physical activity include such as resistance training for muscle strength and bone density, yoga for flexibility and mental well-being, and aerobic activities for cardiovascular health enabling physical therapists’ role. Collaboration between primary care providers, physical therapists, and fitness professionals is essential for developing safe and effective exercise programs for individuals with chronic conditions in order to keep up with the standards of ACSM. However, there is currently a gap in integration between healthcare and fitness systems. Only 17% of general practitioners participate in formal alliances for stimulating physical activity [47]. This interdisciplinary approach ensures that patients receive appropriate guidance, support, and tailored exercise prescriptions to manage their conditions effectively and improve their overall quality of life.

## Limitations

One of the key limitations of this study is not fully represent the broader population’s attitudes or behaviours toward pre-exercise health assessments. Additionally, the study relied on self-reported data, which could introduce biases, as participants might have overestimated or underestimated their engagement in physical activity or health conditions. The cross-sectional design limits the ability to establish causal relationships. Finally, the study was conducted within a specific geographic region (the UAE), which may limit the generalizability of the findings to other regions or populations with different cultural, healthcare, and physical activity contexts. Further research with larger, more diverse samples and longitudinal designs is needed to build a more comprehensive understanding of pre-exercise screening practices.

## Supporting information

S1 Table Participants responses to PARQ+ General health questions. (DOCX)

S2 Table Participants responses to PAR-Q+ follow-up health questions. (DOCX)

S1 File Excel Data of the participants. (XLSX)

## References

[1] Physical activity 2024. Available from: https://www.who.int/news-room/fact-sheets/detail/physical-activity

[2] Mandolesi L, Polverino A, Montuori S, Foti F, Ferraioli G, Sorrentino P, et al. Effects of physical exercise on cognitive functioning and wellbeing: biological and psychological benefits. Front Psychol. 2018;9:509. doi: 10.3389/fpsyg.2018.00509 29755380 PMC5934999

[3] Niemiro GM, Rewane A, Algotar AM. Exercise and fitness effect on obesity. Treasure Island (FL): StatPearls; 2024. 30969715

[4] Warburton DER, Nicol CW, Bredin SSD. Health benefits of physical activity: the evidence. CMAJ. 2006;174(6):801–9. doi: 10.1503/cmaj.051351 16534088 PMC1402378

[5] Dumith SC, Hallal PC, Reis RS, Kohl HW 3rd. Worldwide prevalence of physical inactivity and its association with human development index in 76 countries. Prev Med. 2011;53(1–2):24–8. doi: 10.1016/j.ypmed.2011.02.017 21371494

[6] Katzmarzyk PT. Expanding our understanding of the global impact of physical inactivity. Lancet Glob Health. 2023;11(1):e2–3. doi: 10.1016/S2214-109X(22)00482-X 36480932

[7] Chaabane S, Chaabna K, Abraham A, Mamtani R, Cheema S. Physical activity and sedentary behaviour in the Middle East and North Africa: An overview of systematic reviews and meta-analysis. Sci Rep. 2020;10(1):9363. doi: 10.1038/s41598-020-66163-x 32518254 PMC7283267

[8] Murtagh E, Shalash A, Martin R, Abu Rmeileh N. Measurement and prevalence of adult physical activity levels in Arab countries. Public Health. 2021;198:129–40. doi: 10.1016/j.puhe.2021.07.010 34418764

[9] Dalibalta S, Majdalawieh A, Yousef S, Gusbi M, Wilson JJ, Tully MA, et al. Objectively quantified physical activity and sedentary behaviour in a young UAE population. BMJ Open Sport Exerc Med. 2021;7(1):e000957. doi: 10.1136/bmjsem-2020-000957 33489309 PMC7797257

[10] Alrahma AM, Al Suwaidi H, AlGurg R, Farah Z, Khansaheb H, Ajja R, et al. Results from the United Arab Emirates 2022 report card on physical activity for children and adolescents. J Exerc Sci Fit. 2023;21(2):218–25. doi: 10.1016/j.jesf.2023.02.002 36923208 PMC10009522

[11] DCD. DCD survey reveals 37.7% of respondents achieved WHO’s physical activity recommendations 2023. Available from: https://addcd.gov.ae/Media-Center/News/DCD-Survey-Reveals-of-Respondents-Achieved

[12] Carnethon MR. Physical activity and cardiovascular disease: how much is enough? Am J Lifestyle Med. 2009;3(1 Suppl):44S-49S. doi: 10.1177/1559827609332737 20419076 PMC2857374

[13] Ciampricotti R, Deckers JW, Taverne R, el Gamal M, Relik-van Wely L, Pool J. Characteristics of conditioned and sedentary men with acute coronary syndromes. Am J Cardiol. 1994;73(4):219–22. doi: 10.1016/0002-9149(94)90222-4 8296749

[14] Burke AP, Farb A, Malcom GT, Liang Y, Smialek JE, Virmani R. Plaque rupture and sudden death related to exertion in men with coronary artery disease. JAMA. 1999;281(10):921–6. doi: 10.1001/jama.281.10.921 10078489

[15] Kim JH, Malhotra R, Chiampas G, d’Hemecourt P, Troyanos C, Cianca J, et al. Cardiac arrest during long-distance running races. N Engl J Med. 2012;366(2):130–40. doi: 10.1056/NEJMoa1106468 22236223

[16] Delos D, Maak TG, Rodeo SA. Muscle injuries in athletes: enhancing recovery through scientific understanding and novel therapies. Sports Health. 2013;5(4):346–52. doi: 10.1177/1941738113480934 24459552 PMC3899907

[17] Kokkinos P, Myers J, Doumas M, Faselis C, Manolis A, Pittaras A, et al. Exercise capacity and all-cause mortality in prehypertensive men. Am J Hypertens. 2009;22(7):735–41. doi: 10.1038/ajh.2009.74 19373216

[18] Kokkinos P. Physical activity and cardiovascular disease prevention: current recommendations. Angiology. 2008;59(2 Suppl):26S-9S. doi: 10.1177/0003319708318582 18508850

[19] Liguori G. ACSM’s health-related physical fitness assessment manual. 4th ed. 2018.

[20] Pescatello LS, Franklin BA, Fagard R, Farquhar WB, Kelley GA, Ray CA, et al. American College of Sports Medicine position stand. Exercise and hypertension. Med Sci Sports Exerc. 2004;36(3):533–53. doi: 10.1249/01.mss.0000115224.88514.3a 15076798

[21] SMA. Launch of latest Adult Pre-Exercise Screening System for health and fitness industry. 2019. Available from: https://sma.org.au/launch-of-latest-adult-pre-exercise-screening-system-for-health-and-fitness-industry/

[22] Norton KNL. Exercise And Sports Science Australia, Fitness Australia, Sports Medicine Australia. Pre-Exercise Screening: Guide to the Australian Adult Pre-Exercise Screening System: Exercise And Sports Science Australia; 2011.

[23] Joy EA, Pescatello LS. Pre-exercise screening: role of the primary care physician. Isr J Health Policy Res. 2016;5:29. doi: 10.1186/s13584-016-0089-0 27358724 PMC4926293

[24] Ramamoorthy T, Kulothungan V, Mathur P. Prevalence and correlates of insufficient physical activity among adults aged 18-69 years in India: Findings from the National Noncommunicable Disease Monitoring Survey. J Phys Act Health. 2022;19(3):150–9. doi: 10.1123/jpah.2021-0688 35148500

[25] Abd-Allatif R. Physical activity prevalence among migrants in United Arab Emirates. Journal of Physical Activity and Health. 2019;167.

[26] Herbert WG, Herbert DL, McInnis KJ, Ribisl PM, Franklin BA, Callahan M, et al. Cardiovascular emergency preparedness in recreation facilities at major US universities: college fitness center emergency readiness. Prev Cardiol. 2007;10(3):128–33. doi: 10.1111/j.1520-037x.2007.05708.x 17617775

[27] Perera N, Keyzer P, Dietrich J, Norton K, Sekendiz B, Jones V, et al. Awareness and use of the adult pre-exercise screening system (APSS) in the Australian fitness industry. Br J Sports Med. 2014;48(7):651.2. doi: 10.1136/bjsports-2014-093494.243

[28] Neuhauser HK. The epidemiology of dizziness and vertigo. Neuro-Otology. Handbook of Clinical Neurology. 2016. p. 67–82. 10.1016/B978-0-444-63437-5.00005-4 27638063

[29] Shiozaki T, Ito T, Wada Y, Yamanaka T, Kitahara T. Effects of vestibular rehabilitation on physical activity and subjective dizziness in patients with chronic peripheral vestibular disorders: a six-month randomized trial. Front Neurol. 2021;12:656157. doi: 10.3389/fneur.2021.656157 33995253 PMC8117149

[30] Alsaleh E, Baniyasin F. Prevalence of physical activity levels and perceived benefits of and barriers to physical activity among Jordanian patients with coronary heart disease: A cross-sectional study. Front Public Health. 2023;10:1041428. doi: 10.3389/fpubh.2022.1041428 36684963 PMC9846498

[31] Bytyci Katanolli A, Probst-Hensch N, Ann Obas K, Gerold J, Zahorka M, Jerliu N, et al. Perceived barriers to physical activity behaviour among patients with diabetes and hypertension in Kosovo: a qualitative study. BMC Prim Care. 2022;23(1):257. doi: 10.1186/s12875-022-01866-w 36180857 PMC9523175

[32] Edwards JJ, Coleman DA, Ritti-Dias RM, Farah BQ, Stensel DJ, Lucas SJE, et al. Isometric Exercise Training and Arterial Hypertension: An Updated Review. Sports Med. 2024;54(6):1459–97. doi: 10.1007/s40279-024-02036-x 38762832 PMC11239608

[33] Cornelissen VA. Overcoming barriers to implement exercise in the management of hypertensive patients. Blood Press. 2023;32(1):2208232. doi: 10.1080/08037051.2023.2208232 37143356

[34] (SARC) SRC. UAE National Health Survey Report 2017-2018. Ministry of Health & Prevention; 2018.

[35] Whitfield GP, Pettee Gabriel KK, Rahbar MH, Kohl HW 3rd. Application of the American Heart Association/American College of Sports Medicine Adult Preparticipation Screening Checklist to a nationally representative sample of US adults aged >=40 years from the National Health and Nutrition Examination Survey 2001 to 2004. Circulation. 2014;129(10):1113–20. doi: 10.1161/CIRCULATIONAHA.113.004160 24421370 PMC4094111

[36] Musculoskeletal Health World Health Organization. World Health Organization; 2022. Available from: https://www.who.int/news-room/fact-sheets/detail/musculoskeletal-conditions

[37] Burr J, Shephard R, Cornish S, Vatanparast H, Chilibeck P. Arthritis, osteoporosis, and low back pain: evidence-based clinical risk assessment for physical activity and exercise clearance. Can Fam Physician. 2012;58(1):59–62. 22267624 PMC3264014

[38] Mahmoud I, Saravanan C. Prevalence of mental disorders and the use of mental health services among the adult population in United Arab Emirates. Asian J of Epidemiology. 2019;13(1):12–9. doi: 10.3923/aje.2020.12.19

[39] Noetel M, Sanders T, Gallardo-Gómez D, Taylor P, Del Pozo Cruz B, van den Hoek D, et al. Effect of exercise for depression: systematic review and network meta-analysis of randomised controlled trials. BMJ. 2024;384:e075847. doi: 10.1136/bmj-2023-075847 38355154 PMC10870815

[40] Mahmoud I, Sulaiman N. Prevalence of metabolic syndrome and associated risk factors in the United Arab Emirates: a cross-sectional population-based study. Front Public Health. 2022;9:811006. doi: 10.3389/fpubh.2021.811006 35141192 PMC8818742

[41] Federation ID. IDF Diabetes Atlas. 10th ed. Belgium: International Diabetes Federation; 2021.

[42] American Diabetes Association. 5. lifestyle management: standards of medical care in diabetes-2019. Diabetes Care. 2019;42(Suppl 1):S46–60. doi: 10.2337/dc19-S005 30559231

[43] Services USDoHaH. Physical Activity Guidelines for Americans. 2nd ed. U.S. Department of Health and Human Services; 2018.

[44] Armstrong M, Paternostro-Bayles M, Conroy MB, Franklin BA, Richardson C, Kriska A. Preparticipation screening prior to physical activity in community lifestyle interventions. Transl J Am Coll Sports Med. 2018;3(22):176–80. doi: 10.1249/TJX.0000000000000073 30873436 PMC6411298

[45] American College of Sports Medicine, Liguori G, Feito Y, Fountaine C, Roy B. ACSM’s guidelines for exercise testing and prescription. 11th ed. Philadelphia: Wolters Kluwer; 2022. xxxiv, 513 p.

[46] Arietaleanizbeaskoa MS, Sancho A, Olazabal I, Moreno C, Gil E, Garcia-Alvarez A, et al. Effectiveness of physical exercise for people with chronic diseases: the EFIKRONIK study protocol for a hybrid, clinical and implementation randomized trial. BMC Fam Pract. 2020;21(1):227. doi: 10.1186/s12875-020-01298-4 33158422 PMC7648284

[47] Leemrijse CJ, de Bakker DH, Ooms L, Veenhof C. Collaboration of general practitioners and exercise providers in promotion of physical activity a written survey among general practitioners. BMC Fam Pract. 2015;16:96. doi: 10.1186/s12875-015-0316-8 26245953 PMC4527276

