# Supplementary material for: Pre-exercise health screening in the UAE: A necessity or barrier to engage in physical activity?
Source: PLoS One. 2025 May 30;20(5):e0325246. doi: 10.1371/journal.pone.0325246 (PMC12124491; doi:10.1371/journal.pone.0325246)
Supplement: S1 Table — (DOCX) [file pone.0325246.s001.docx]

**S1 Table:** Participants responses to PARQ+ General health questions

| **General Health Questions** | | No. | % |
| --- | --- | --- | --- |
| Has your doctor ever said that you have a heart condition OR high blood pressure? | No | 546 | 86.7 |
|  | Yes | 84 | 13.3 |
| Do you feel pain in your chest at rest, during your daily activities of living, OR when you do physical activity? | No | 532 | 84.4 |
|  | Yes | 98 | 15.6 |
| Do you lose balance because of dizziness OR have you lost conscious in the last 12 months? | No | 514 | 81.6 |
|  | Yes | 116 | 18.4 |
| Have you ever been diagnosed with another chronic medical condition (Other than heart disease or high blood pressure)? | No | 556 | 88.3 |
|  | Yes | 74 | 11.7 |
| Are you currently taking prescribed medications for a chronic medical condition? | No | 562 | 89.2 |
|  | Yes | 68 | 10.8 |
| Do you currently have (or have had within past 12 months) a bone, joint, or soft tissue (muscle, ligament, or tendon) problem that could made worse by becoming more physically active? | No | 530 | 84.1 |
|  | Yes | 100 | 15.9 |
| Has your doctor ever said that you should only do medically supervised physical activity? | No | 574 | 91.1 |
|  | Yes | 56 | 8.9 |
