# Supplementary material for: Pre-exercise health screening in the UAE: A necessity or barrier to engage in physical activity?
Source: PLoS One. 2025 May 30;20(5):e0325246. doi: 10.1371/journal.pone.0325246 (PMC12124491; doi:10.1371/journal.pone.0325246)
Supplement: S2 Table — (DOCX) [file pone.0325246.s002.docx]

**S2 Table:** Participants responses to PAR-Q+ follow-up health questions

| **Follow Up Questions (Further evaluation)** | | No. | % |
| --- | --- | --- | --- |
| Do you have Arthritis, Osteoporosis, or Back Problems? | Yes | 64 | **11.4** |
|  | No | 496 | 88.6 |
| Do you currently have Cancer of any kind? | Yes | 22 | 3.9 |
|  | No | 538 | 96.1 |
| Do you currently have High Blood Pressure? | Yes | 25 | 4.5 |
|  | No | 535 | 95.5 |
| Do you have a Heart or Cardiovascular Condition? This includes Coronary Artery Disease, Heart Failure, Diagnosed Abnormality of Heart Rhythm | Yes | 24 | 4.3 |
|  | No | 536 | 95.7 |
| Do you have any Metabolic Conditions? This includes Type 1 Diabetes, Type Diabetes, Pre- Diabetes | Yes | 34 | **6.1** |
|  | No | 526 | 93.9 |
| Do you have any Mental Health Problems or Learning Difficulties? | Yes | 38 | **6.8** |
|  | No | 522 | 93.2 |
| Have you had a Stroke? This includes Transient Ischemic Attack (TIA) or Cerebrovascular Accident | Yes | 19 | 3.4 |
|  | No | 541 | 96.6 |
| Do you have a respiratory disease? | Yes | 29 | 5.2 |
|  | No | 531 | 94.8 |
| Do you have a Spinal Cord Injury? | Yes | 22 | 4.2 |
|  | No | 496 | 95.8 |
| Do you have any other medical condition not listed above or do you have two or more medical conditions? | Yes | 43 | **7.7** |
|  | No | 517 | 92.3 |
